# Classification of gallbladder cancer by assessment of CD8^+^ TIL and PD-L1 expression

**DOI:** 10.1186/s12885-018-4651-8

**Published:** 2018-07-28

**Authors:** Jianzhen Lin, Junyu Long, Xueshuai Wan, Jingci Chen, Yi Bai, Anqiang Wang, Xiaobo Yang, Yan Wu, Simon C. Robson, Xinting Sang, Haitao Zhao

**Affiliations:** 1 Department of Liver Surgery, Chinese Academy of Medical Sciences and Peking Union Medical College (CAMS & PUMC), Peking Union Medical College Hospital, #1 Shuaifuyuan, Wangfujing, Beijing, 100730 China; 2 Liver Center and The Transplant Institute, Department of Medicine, Beth Israel Deaconess Medical Center, Harvard Medical School, Boston, MA USA; 3 School of Medicine, Tsinghua University, Beijing, China; 4 Department of Gastrointestinal Surgery, Peking University Cancer Hospital & Institute, Beijing, China; 5 Center of Translational Medicine, Chinese Academy of Medical Sciences and Peking Union Medical College, Peking Union Medical College Hospital, Beijing, China

**Keywords:** Gallbladder cancer, Immunohistochemistry, Immune microenvironment, PD-L1, CD8^+^ TILs

## Abstract

**Background:**

Programmed death ligand 1/2 (PD-L1/PD-L2) expression has been established as a prognostic factor for various solid tumors and as a predictive factor for PD-1 blockade therapy, but scant data on its role in gallbladder cancer (GBC). The aims of this study were to assess the expression of PD-L1/PD-L2 and the density of CD8^+^ tumor-infiltrating lymphocytes (TIL) from GBC samples and to quantify the association between survival prognosis and these factors.

**Methods:**

CD8^+^ TILs density and the expression of PD-1, PD-L1, PD-L2 and CD133 were assessed using immunohistochemistry in tumor specimens from 66 patients with gallbladder adenocarcinoma. These indexes were correlated with the clinicopathological features.

**Results:**

The rate of PD-L1-positive (PD-L1^+^) was 54%, which included 18% positivity in tumor cells, and 36% in peritumoral immune stroma. High CD8^+^ TIL density (CD8^high^) was observed in PD-L1^+^ GBC, and PD-L1^+^ was positively associated with PD-L2^+^ expression. Regarding prognostic factors, PD-L1^+^ expression was related to worse overall survival (OS), and CD8^high^ indicated better OS and progression-free survival (PFS). The combination of CD8^high^ with PD-L1^+^ serves as a prognostic factor for improved OS (*P* < 0.001) and PFS (*P* = 0.014).

**Conclusion:**

Analysis of the tumor immune microenvironment based on CD8^+^ TIL and PD-L1 expression is a promising independent predictor for the clinical outcome of GBC patients.

**Electronic supplementary material:**

The online version of this article (10.1186/s12885-018-4651-8) contains supplementary material, which is available to authorized users.

## Background

As a relatively rare cancer among Western populations, gallbladder cancer (GBC) is more prevalent in Southeast Asia and Chile [1]. Although its incidence is low, mortality due to GBC is relatively high, and the prognosis is poor [2]. The primary risk factors for GBC are gallstones, gallbladder polyps, infection, diabetes [3] and porcelain gallbladder [4]. A satisfactory prognosis of GBC depends on an early diagnosis and completed resection. However, because the early stages are asymptomatic, most GBC are discovered at clinical late or metastatic stages. Therefore, fewer than 10% of patients are eligible for curative surgery, and more than half of GBC present lymph node metastasis [5]. After surgery, most patients with GBC develop to recurrent and metastatic disease [6].

Immunotherapy has presented a marginal therapeutic option in cancer in the past two decades [7]. Recently, immune checkpoint inhibitors that target the programmed death receptor 1/ligand 1 (PD-1/PD-L1) have displayed promising antitumor effects in different types of solid tumors [8–10]. Various researches have confirmed that PD-L1 induces T-cell immune suppression and therefore favors tumor progression [11]; thus, expression status of PD-L1 served as a prognostic factor in various types of tumor. Moreover, immunohistochemical (IHC) evaluation of PD-L1 is thought to represent a viable method to predict PD-1 inhibitor sensitivity. PD-L2, the second of PD-1 ligand, had the ability to aggressively inhibit T cell receptor (TCR)-mediated proliferation and cytokine production by CD4^+^ T cells through combination with PD-1 in a mouse model [12]. More significantly, PD-L2 expression was reported to be strongly correlated with PD-1 inhibitor outcome [13].

Cytotoxic T lymphocytes (CTLs), an crucial role in immune responses to cancers, can recognize tumor cells in an antigen-specific manner, which primarily results from the abundant expression of several tumor associated antigens (TAAs) [14]. Thus, it is essential to assess the expression of CD8+ tumor-infiltrating lymphocytes (TILs). Moreover, CD133, a membranous surface protein, was reported to have a negative correlation with GBC patients’ prognosis [15]. Additionally, CD133^+^ GBC cells exhibited highly resistance to conventional chemotherapy. Therefore, PD-1/L1 expression status among CD133^+^ GBC patients deserves to explore to expand the possibility of PD-1 inhibitor treatment [16].

Throughout the published literatures, scant information has been reported on the expression levels of PD-1/PD-L1/PD-L2 in GBC and their correlations with the clinicopathological features of GBC and the CD8^+^ TIL status. Thus this study sought to characterize the expression of PD-1 and its ligands PD-L1/PD-L2 in a series of 66 formalin-fixed, paraffin-embedded (FFPE) gallbladder adenocarcinoma specimens and to associate these expression levels with various underlying risk factors. We also explored the relationships between immune checkpoint markers and both the tumor immune microenvironment (CD8^+^ TILs) and progenitor-like tumor cells (TCs) (CD133^+^).

## Methods

### Specimens and patients

FFPE tissues from primary GBC lesions with adenocarcinoma were obtained from 66 patients at Peking Union Medical College Hospital (PUMCH) between 2009 and 2014. GBC was confirmed histopathologically by gastroenterology (GI) pathologists according to the American Joint Committee on Cancer (AJCC) cancer staging system (7th edition) and the WHO classification systems. This study was approved by the local ethics committee at PUMCH, and written consent was obtained from all enrolled patients.

The following clinical and biological features were systematically collected from the PUMCH electronic medical records: patient age, gender, risk factors (e.g., gallstone, cholecystitis, diabetes, and hypertension), preoperative serum tests (liver function indexes, CEA and CA19–9), margin of tumor resection, histologic grade, TNM stage, tumor lesion size, lymph node involvement, vascular invasion by tumor, progression-free survival (PFS) and overall survival (OS).

### Follow-up arrangement

Follow-up was completed on April 18, 2017, Follow-up with median of 25 months (range: 3–65 months). Disease progression events were defined as progressive changes in the typical imaging appearance on CT and/or MRI, according to Response Evaluation Criteria in Solid Tumors (RECIST) version 1.1 [17]. OS was the interval either between initial diagnosis and death or between initial diagnosis and the last observation for surviving patients. PFS was the length of time between treatment (surgery) and the occurrence of disease progression events. Data were censored at the last follow-up for living patients.

### Immunohistochemical (IHC) staining and evaluation

Immunostaining was performed on FFPE specimens [18]. Serial 4-μm-thick sections were sliced and placed onto glass slides for IHC staining. The following primary antibodies were used: anti-PD-1 (mouse monoclonal NAT105, dilution: 1/50, Abcam, Shanghai, China); anti-PD-L1 (rabbit monoclonal E1L3N, dilution:1/100, Cell Signaling Technology, Danvers, MA); anti-PD-L2 (mouse monoclonal Clone#176611, dilution: 1/100, R&D Systems, Minneapolis); anti-CD8 (mouse monoclonal 4B11, dilution: 1/50, Invitrogen, US); and anti-CD133 (rabbit polyclonal ab16518, dilution: 1/100, Abcam, Cambridge, MA, USA). Secondary antibodies were added to all the sections, including negative control slides (which omitted the primary antibody treatment).

Evaluation of protein expression was performed by two independent investigators who were blinded to the clinicopathologic data. Opposing results were re-evaluated by the same investigators, who remained blinded to the clinicopathologic data and the other investigator’s opinion. If a consensus still could not be reach after the re-evaluation, a third independent pathologist who was also blinded to the clinicopathologic data performed an evaluation. The majority (two out of three) diagnosis was recorded. The eventual result was approved by at least two pathologists, and a consensus decision was reached.

### Evaluation of immunohistochemical variables

A computerized image analysis system was used to evaluate density of the CD8^+^ TILs, which was comprised by a Leica DFC495 Digital Color Microscope Camera installed on a Leica DMLA light microscope (Leica Microsystems, Wetzlar, Germany) and linked to a computer. Within 400× magnification, there existed at least 8 independent and intact microscopic intratumoral fields for each slide. Five unique microscopic fields (400×) were randomly chose for each patient sample to warrant representativeness. The results were expressed as the mean (±SE) number cells per computerized 400× microscopic field (0.09975 mm^2^/field) [19].

PD-L1 evaluation in both GBC TCs and the peritumoral immune stroma (IS) included TILs and tumor-associated macrophages (TAMs). Samples containing ≥5% expression in any TCs were considered PD-L1 positive in TCs (PD-L1^+^ TCs). Any samples with > 1% PD-L1 expression in TILs and TAMs and simultaneous expression (< 5% or negative) of PD-L1 in TCs were defined as PD-L1 positive only in IS (PD-L1^+^ IS). The PD-L1-positive group (PD-L1^+^) included PD-L1-positive expression in TCs and IS, whereas all other samples were classified as the PD-L1-negative group (PD-L1^−^).

For CD133, positive expression was defined as ≥5% expression in TCs. For PD-L2, positive expression was defined as either > 5% in TCs or > 1% positive in peritumoral IS. Moreover, PD-1 expression was observed only in intratumoral lymphocytes, and ≥ 1% in TILs expression was defined as PD-1-positive.

### Statistical analysis

SPSS software version 24.0 (SPSS; IBM, Chicago, IL, USA) was used to perform statistical analysis. Continuous variables and proportions were compared using the chi-squared, Fisher’s exact tests, and Mann-Whitney as appropriate. Correlation between marker expression levels was analyzed by the Spearman rank correlation coefficient. Univariate and multivariate analyses were based on the Cox proportional hazards regression model. In assessing the density of CD8^+^ TILs, the cut-off for stratifying subgroups was the median value. A two-tailed *P* < 0.05 was considered statistically significant.

## Results

### Patients characteristics and clinical and pathological features

The main clinical characterizes of the 66 GBC patients are listed in Additional file 1: Table S1. The patients were primarily females (38/66, 58%), and the median age was 65 years (range: 29–81). Risk factors included gallstone (*n* = 36, 55%), diabetes (*n* = 20, 30%), hypertension (*n* = 25, 38%) and cholecystitis (*n* = 43, 65%).

Tumor stage was divided into two classes according to the AJCC 7th edition cancer staging system, in which the TNM stage was I + II (*n* = 22, 33%) or III + IV (*n* = 44, 67%, 4 cases were stage IV). A total of 70% (46/66) GBC patients had lymph node metastasis. Elevated CEA levels (> 5 μg/L) were detected in 30% (20/66) of patients, and abnormal CA19–9 levels (> 34 U/mL) were found in 53% (35/66) of patients. The initial diagnosis symptom of jaundice was observed in 21% of patients (14/66).

Tumor slides containing high-level fibrotic, hemorrha-gic and necrotic components were excluded through hematoxylin and eosin (HE) staining. Poorly differentiated tumors were prevailing (58% [38/66] of cases). The detailed pathological features are described in Additional file 1: Table S1.

Over a median follow-up time of 25 months (2.1 years, range 3–65 months), 6 patients (9%) were lost to follow-up, 51 patients (77%) died, and 4 patients (6%) were still disease progression-free; these data corresponded to a 1-year OS rate of 59% (95% CI: 47–71%) and a 2-year OS rate of 38% (95% CI: 25–50%).

### Expression of PD-L1 and PD-L2 and CD8^+^ TIL density in GBC

**Fig. 1:**
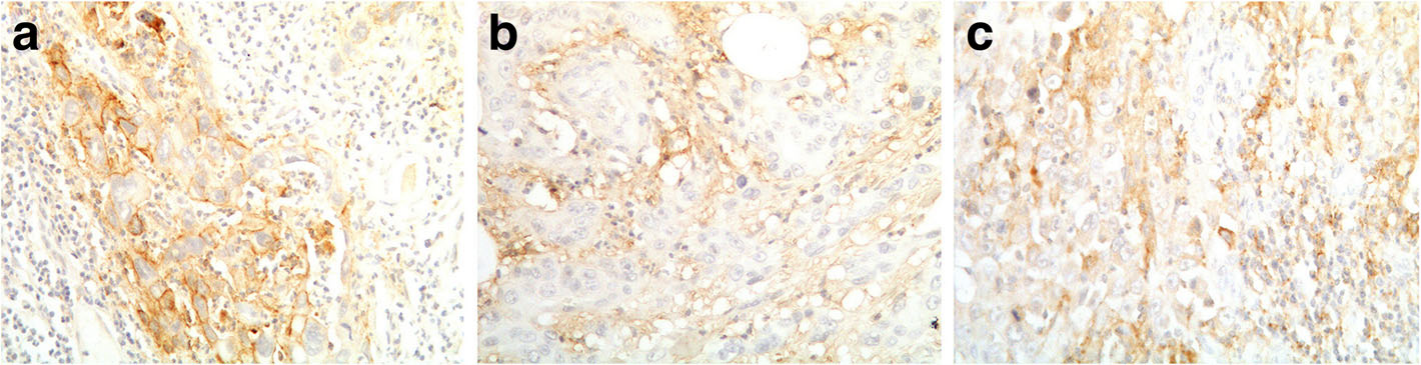
Expression of PD-L1 by gallbladder cancer (GBC) (× 200 magnification). Representative examples are shown of programmed death-ligand 1 (PD-L1) immunohistochemical staining of samples that are PD-L1-positive only in tumor cells (PD-L1^+^ TCs, **a**), PD-L1-positive only in immune stroma (PD-L1^+^ IS, **b**) and positive for PD-L1 expression (PD-L1^+^, **c**)

We observed two patterns of PD-L1-positive expression: positive in the membrane of TCs and positive in the peritumor IS (Fig. 1a). A subset of GBC patients (18%, 12/66, 95% CI: 8.6–27.7%) exhibited PD-L1^+^ TCs. Intriguingly, all 12 cases showed > 1% PD-L1-positive in the IS (Fig. 1b). Another 36% (24/66, 95% CI: 24.4–48.3%) of patients were PD-L1^+^ IS, in which PD-L1 was positive (> 1%) in the IS but negative (< 5%) in TCs. Overall, PD-L1-positive expression (PD-L1^+^ group, Fig. 1c) accounted for 54% of all patients (36/66, 95% CI: 42.4–66.9%).

Regarding PD-L2 expression in GBC tumor tissue, 67% (44/66, 95% CI: 55.0–78.3%) of the specimens exhibited PD-L2-positive expression (> 5% positive in TCs and > 1% positive in peritumoral IS). In our cohort of patients with GBC, PD-L2-positive expression was more common than PD-L1^+^ expression.

CD8^+^ TIL density was quantified in all the GBC samples. The median CD8^+^ TIL density was 46 cells/field (range 4–275). The associations between the PD-L1^+^ TCs, PD-L1^+^ IS and PD-L1^−^ groups and the clinicopathological features of the GBC patients are summarized in Additional file 1: Table S2. We found that PD-L1^+^ GBC showed a higher probability of positive PD-L2 expression in tumor tissues (83% vs. 47%, *P* = 0.002) and a 1.54-fold increase in the median CD8^+^ TIL density (PD-L1^+^ vs. PD-L1^−^: 53/field vs. 34.5/field, *P* = 0.029), whereas no difference in density of the CD8^+^ TILs existed between PD-L1^+^ TCs and PD-L1^+^ IS (47.5% vs. 57%, *P* = 0.568).

### Association between CD8^+^ TILs and clinicopathological parameters

Using the median value of CD8^+^ TIL density (46/field) as a cut-off, we divided the 66 patients with GBC into a CD8^high^ TILs cluster (*n* = 33, 50%) and a CD8^low^ TILs cluster (*n* = 33, 50%). The CD8^+^ TIL densities in these subgroups showed a significant discrepancy, with 74/field in the CD8^high^ cluster and 27/field in the CD8^low^ cluster (*P* < 0.001). The comparison of the CD8^high^ and CD8^low^ groups regarding the clinicopathological characteristics of GBC is summarized in Additional file 1: Table S2. No significant differences were observed in clinicopathological factors between the CD8^high^ and CD8^low^ TILs.

### Expression of progenitor-like biomarker and clinicopathological features

We selected CD133 as a progenitor-like biomarkers. Briefly, CD133 expression was observed only in the membranes of TCs (Additional file 1: Figure S1), with a positive expression rate of 41% (27/66, 95% CI: 29–53%). However, CD133 expression was not correlated with PD-L1 expression, CD8^+^ TIL density, or post-operative survival (PFS and OS, Additional file 1: Figure S2).

### Prognostic significance of PD-L1 expression, CD8^+^ TIL density and classification based on combined CD8^+^ TILs and PD-L1 expression attributes

**Fig. 2:**
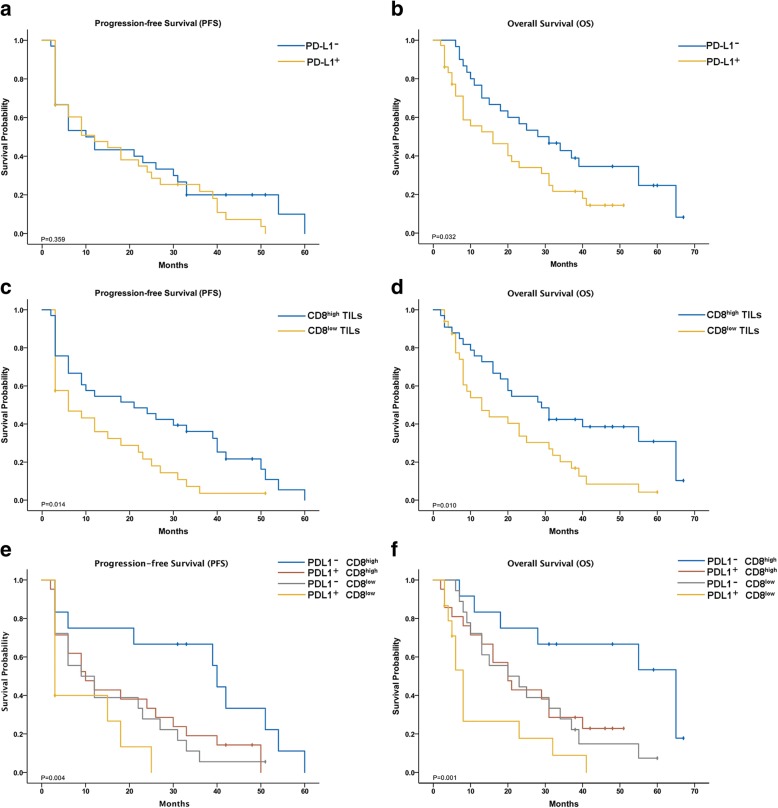
Kaplan-Meier analysis of progression-free survival and overall survival according to PD-L1 expression (**a** and **b**), CD8^+^ tumor-infiltrating lymphocytes (TILs) (**c** and **d**), and classification based on combining PD-L1 expression and CD8^+^ TILs

As mentioned above, the PD-L1 expression pattern included PD-L1^+^ TCs, PD-L1^+^IS, and PD-L1^−^. Using Kaplan-Meier survival analysis, we found that PD-L1^+^ TCs was weakly associated with worse PFS (*P* = 0.042) but not with OS (*P* = 0.058, Additional file 1: Figure S3A-B), and there was no significant association among these three patterns (Additional file 1: Figure S3C-D). The PD-L1^+^ group, which included PD-L1^+^ TCs and PD-L1^+^IS, was related only with prognosis for OS and had no influence on PFS. The median OS for GBC patient with PD-L1^+^ was significantly more than that for patients with PD-L1^−^ (20.5 vs. 34.2 months, respectively; *P* = 0.032, Fig. 2a), while the difference in PFS time was nonsignificant (15.2 vs. 23.6 months, respectively; *P* = 0.062, Fig. 2b).

Longer PFS and OS were observed in the CD8^high^ group than in the CD8^low^ group (24.6 vs. 13.2 months, respectively; *P* = 0.014, Fig. 2c; 34.9 vs. 20.2 months, respectively; *P* = 0.01, Fig. 2d). Therefore, CD8^+^ TIL density around tumor was a prognostic factor for both OS and PFS.

**Fig. 3:**
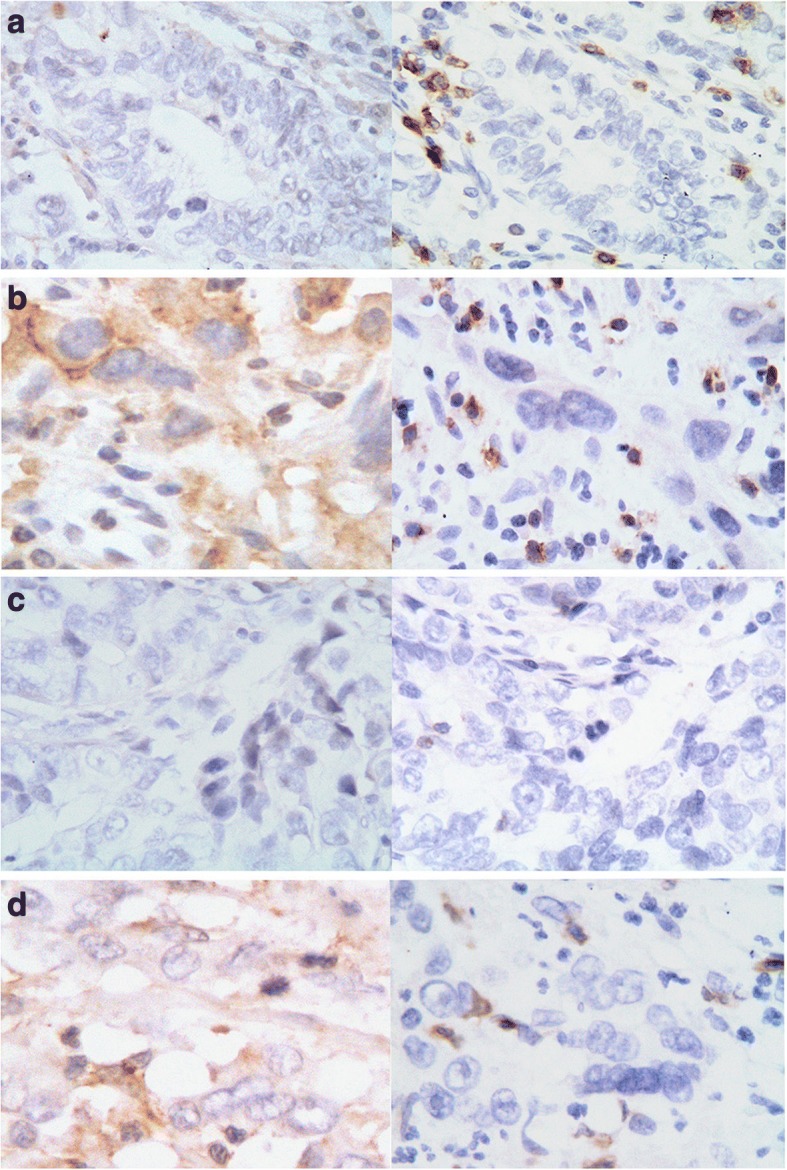
Representative staining patterns of samples classified through combining PD-L1 expression and CD8^+^ TILs (× 400 magnification). The left panel shows the expression of PD-L1 and the right panel shows the status of CD8^+^ TILs. I: PD-L1^−^ CD8^high^ (**a**); II: PD-L1^+^ CD8^high^ (**b**); III: PD-L1^−^ CD8^low^ (**c**); IV: PD-L1^+^ CD8^low^ (**d**)

Importantly, we stratified our cohort into four groups through the combined evaluation of PD-L1 expression and CD8^+^ TIL density: I, PD-L1- and CD8^high^ (*n* = 12); II, PD-L1^+^ and CD8^high^ (*n* = 21); III, PD-L1^−^ and CD8^low^ (*n* = 18); and IV, PD-L1^+^ and CD8^low^ (*n* = 15) (Fig. 3a-d). Among the four groups, there were significant differences in both OS (*P* = 0.001) and PFS (*P* = 0.004) (Fig. 2e-f), wherein the patients with PD-L1^−^ and CD8^high^ had the best survival, and the patients with PD-L1^+^ and CD8^low^ TILs had the worst survival.

### Prognostic factors

In the univariate analysis by Cox regression model, age, sex, common risk factors, AST and TBil levels, tumor size, PD-L1^+^ TCs and the expression levels of CD133, PD-1 and PD-L2 showed no prognostic significance regarding OS or PFS (Table 1 and Additional file 1: Figure S2A-F). For both PFS and OS, the significant prognostic factors included an advanced TNM stage; jaundice; completed resection; elevated serum levels of CA19–9, ALT, DBil and GGT; CD8^+^ TIL density and classification based on combining PD-L1 expression and CD8^+^ TILs. Specifically, elevated serum levels of CEA and ALP, tumor differentiation status and PD-L1^−^ expression were associated with OS (Table 1).

**Table 1:** Univariate analysis factors associated with PFS or OS

	OS	PFS
	HR (95% CI) *P*-value	HR (95% CI) *P*-value
Clinical parameters		
Age, (≤ 65 v > 65)	1.50 (0.84–2.67) *P* = 0.170	1.39 (0.81–2.39) *P* = 0.230
Sex, (Male v Female)	1.18 (0.68–2.06) *P* = 0.559	1.061 (0.66–1.81) *P* = 0.829
TNM stage, (I + II vs. III + IV)	2.76 (1.45–5.24) *P* = 0.002	2.10 (1.18–3.73) *P* = 0.012
Risk factors		
Gall stone (no v yes)	1.58 (0.89–2.81) *P* = 0.117	1.47 (0.85–2.54) *P* = 0.166
Diabetes (no v yes)	1.27 (0.66–2.45) *P* = 0.467	1.15 (0.64–2.05) *P* = 0.640
Hypertension (no v yes)	1.40 (0.77–2.52) *P* = 0.270	1.50 (0.86–2.63) *P* = 0.156
Cholecystitis (no v yes)	1.34 (0.74–2.41) *P* = 0.335	1.34 (0.77–2.35) *P* = 0.304
Jaundice (no v yes)	2.82 (1.49–5.33) *P* = 0.001	2.58 (1.35–4.92) *P* = 0.004
Completed resection (no v yes)	0.38 (0.21–0.68) *P* = 0.001	0.40 (0.22–0.72) *P* = 0.002
CEA (> 5 μg/L)	1.97 (1.08–3.60) *P* = 0.028	1.61 (0.90–2.89) *P* = 0.110
CA19–9 (> 34 U/mL)	2.77 (1.52–5.06) *P* = 0.001	2.28 (1.28–4.04) *P* = 0.005
Liver dysfunction		
ALT (> 40 U/L)	2.13 (1.15–3.95) *P* = 0.016	2.04 (1.10–3.79) *P* = 0.023
AST (> 35 U/L)	1.63 (0.91–2.91) *P* = 0.099	1.34 (0.79–2.42) *P* = 0.264
TBil (> 22.2 μmol/L)	1.625 (0.91–2,90) *P* = 0.101	1.38 (0.79–2.44) *P* = 0.261
DBil (> 6.8 μmol/L)	2.71 (1.50–4.91) *P* = 0.001	2.45 (1.35–4.47) *P* = 0.003
GGT (> 45 U/L)	2.34 (1.31–4.17) *P* = 0.004	2.055 (1.17–3.61) *P* = 0.013
ALP (> 135 U/L)	1.99 (1.12–3.56) *P* = 0.020	1.75 (0.99–3.08) *P* = 0.055
Pathological features		
Differentiation (moderate vs. poor)	1.95 (1.10–3.46) *P* = 0.023	1.57 (0.92–2.68) *P* = 0.100
Mean tumor size (≤ 3.0 v > 3.0 cm)	1.07 (0.61–1.88) *P* = 0.816	1.02 (0.60–1.72) *P* = 0.955
Immunohistochemistry		
CD133^+^ in TCs	1.09 (0.62–1.91) *P* = 0.776	0.99 (0.58–1.71) *P* = 0.994
CD8^+^ TILs (CD8^low^ vs. CD8^high^)	0.49 (0.28–0.86) *P* = 0.014	0.54 (0.31–0.93) *P* = 0.026
PD-1^+^ in TILs	1.14 (0.66–1.98) *P* = 0.636	1.26 (0.74–2.13) *P* = 0.398
PD-L1^+^ in TCs	1.92 (0.95–3.88) *P* = 0.067	1.87 (0.95–3.68) *P* = 0.071
PD-L1^−^ vs. PD-L1^+^	1.86 (1.04–3.33) *P* = 0.038	1.60 (0.93–2.75) *P* = 0.088
PD-L2- vs. PD-L2^+^	1.52 (0.82–2.80) *P* = 0.185	1.40 (0.80–2.46) *P* = 0.242
Classification based on PD-L1 expression and CD8^+^ TILs		
Overall	NA; *P* = 0.003	NA; *P* = 0.018
I vs. II	3.14 (1.11–8.85) *P* = 0.031	2.40 (1.03–5.59) *P* = 0.042
I vs. III	3.36 (1.21–9.30) *P* = 0.020	2.75 (1.16–6.51) *P* = 0.021
I vs. IV	7.38 (2.49–21.89) < 0.001	4.81 (1.81–12.78) *P* = 0.002

NOTE: Univariate analysis, Cox proportional hazards regression model

The multivariate Cox proportional hazards analyses included parameters were significant in the univariate analysis (based on Wald forward selection) (Table 2). Completed resection (R0 surgery), which is often considered a prognostic predictor for GBC, was an independent prognostic factor for PFS in our cohort. Advanced TNM stage and completed resection remained associated with OS and were individual independent prognostic factors for OS. Importantly, a high density of CD8^+^ TILs in combination with negative PD-L1 expression was an independent factor for prolonged OS (*P* = 0.002) and improved PFS (*P* = 0.014). We demonstrated that co-assessment of CD8^+^ TILs and PD-L1 expression has prominent prognostic significance for OS among patients with GBC.

**Table 2:** Multivariate analysis factors associated with PFS or OS

	Hazard Ratio	95% CI	*P*-value
Overall survival (OS)			
TNM stage (I + II vs. III + IV)	2.29	1.14–4.58	0.019
Completed resection (no v yes)	0.36	0.17–0.75	0.006
Classification based on PD-L1 expression and CD8^+^ TILs			
Overall	NA	NA	< 0.001
I vs. II	3.71	1.32–10.41	0.013
I vs. III	2.06	0.72–5.85	0.177
I vs. IV	9.60	3.20–28.84	0.001
Progression-free survival (PFS)			
Completed resection (no v yes)	0.40	0.22–0.72	0.002
Classification based on PD-L1 expression and CD8^+^ TILs			
Overall	NA	NA	0.014
I vs. II	2.61	1.12–6.09	0.027
I vs. III	2.15	0.89–5.20	0.090
I vs. IV	4.99	1.87–13.31	0.001

Note: Multivariate analysis, Cox proportional hazards regression model (based on Wald Forward selection)

Variables were adopted for their prognostic significance by univariate analysis

Subgroups: I (CD8^high^&PD-L1^−^); II (CD8^high^&PD-L1^+^); III (CD8^low^&PD-L1^−^); IV (CD8^low^&PD-L1^+^)

### Clinicopathological parameters and treatment among the four subgroups

Table 3 summarizes the clinicopathological parameters of four subgroups derived from the classification based on CD8^+^ TIL density and PD-L1 expression. No clinicopathological parameters exhibited significant diversity among the four subgroups.

**Table 3:** Relationship of four classes based on PD-L1 expression and CD8^+^ TILs

	Subgroup I	Subgroup II	Subgroup III	Subgroup IV
	CD8^high^ PD-L1^−^	CD8^high^ PD-L1^+^	CD8^low^ PD-L1^−^	CD8^low^ PD-L1^+^
Clinical parameters n (%)	(*n* = 12)	(*n* = 21)	(*n* = 18)	(*n* = 15)
Age median (range)	65 (50–79)	64 (29–75)	66 (48–79)	70 (45–81)
Sex (Female)	7 (58%)	12 (57%)	10 (56%)	9 (60%)
TNM stage (III + IV)	7 (58%)	11 (52%)	14 (78%)	11 (73%)
Risk factors				
Gallstone	3 (25%)	13 (62%)	11 (61%)	9 (60%)
Diabetes	4 (33%)	6 (29%)	7 (39%)	3 (20%)
Hypertension	4 (33%)	8 (38%)	8 (44%)	5 (33%)
Cholecystitis	7 (58%)	12 (57%)	14 (78%)	10 (67%)
Jaundice	0 (0%)	6 (29%)	4 (22%)	4 (27%)
Completed resection	8 (67%)	16 (76%)	11 (61%)	11 (73%)
CEA (> 5 μg/L)	2 (17%)	5 (24%)	4 (22%)	9 (60%)
CA19–9 (> 34 U/mL)	4 (33%)	13 (62%)	11 (61%)	7 (58%)
Liver dysfunction				
ALT (> 40 U/L)				
AST (> 35 U/L)	1 (8%)	5 (24%)	5 (28%)	4 (27%)
TBil (> 22.2 μmol/L)	3 (25%)	5 (24%)	6 (33%)	5 (33%)
DBil (> 6.8 μmol/L)	3 (25%)	7 (33%)	5 (28%)	5 (33%)
GGT (> 45 U/L)	1 (8%)	7 (33%)	5 (28%)	5 (33%)
ALP (> 135 U/L)	3 (25%)	7 (33%)	5 (28%)	5 (33%)
	2 (17%)	9 (43%)	5 (28%)	4 (27%)
Pathological features				
Differentiation (Poor)	5 (42%)	12 (57%)	9 (50%)	12 (80%)
Tumor size, cm (median, range)	2.4 (0.6–7.5)	3.5 (0.5–7.0)	3.0 (0.6–6.0)	2.0 (0.8–7.0)
Immunohistochemistry				
CD133^+^ in TCs, n (%)	5 (42%)	9 (43%)	6 (33%)	7 (58%)
PD-1^+^ in TILs, n (%)	7 (58%)	9 (43%)	6 (33%)	11 (73%)
PD-L2^+^ in TCs, n (%)	5 (42%)	17 (81%)	9 (50%)	13 (87%)
CD8+ TILs density	60.5	79	28	27
(median, range)	(47–167)	(49–275)	(4–42)	(8–46)
Adjuvant treatment				
Chemotherapy, n (%)	7 (58%)	12 (57%)	13 (72%)	8 (53%)
Radiotherapy, n (%)	2 (17%)	5 (24%)	4 (22%)	4 (27%)
Others, n (%)	3 (25%)	8 (33%)	6 (33%)	4 (27%)
No, n (%)	2 (17%)	4 (19%)	2 (11%)	2 (13%)
Palliative treatment at stage IV				
With therapy, n (%)	8 (67%)	16 (76%)	15 (83%)	12 (80%)
Without therapy, n (%)	4 (33%)	5 (24%)	3 (17%)	3 (20%)

Adjuvant therapies were administered to all 66 GBC patients. No patients in our cohort received any line treatments of immunotherapies, such as immune-checkpoint inhibitors or CAR-T cell therapies. No significant difference in post-progression treatment approaches was observed among the different subgroups (Table 3).

## Discussion

Immune checkpoint inhibitors targeting the PD-1/PD-L1 pathway have exhibited potent efficacy in various solid malignancies. Clinical benefits were strongly correlated with PD-L1 expression in tumors assessed using IHC [20]. Moreover, PD-L1 expression has been demonstrated to be an important prognostic factor in several types of cancer. However, no studies have evaluated PD-L1 expression and its clinical significance in GBC patients.

The current work provides evidence that GBC patients mount a T-cell mediated immune response against TCs via the PD-1 pathway. In a cohort of 66 tumor specimens from GBC patients, 18% of the GBC samples exhibited PD-L1^+^ expression in TC membrane. By contrast, 36% of the GBC samples exhibited PD-L1 expression only in the IS. Overall, 54% of GBCs were PD-L1^+^. The PD-L2^+^ expression rate was 67% in the GBC samples and we found that PD-L2^+^ expression was positively associated with PD-L1 expression (*P* = 0.002), indicating possible co-expression of PD-L1 and PD-L2 (Additional file 1: Figure S4). PD-L1 positive alone was not correlated with any clinicobiological or pathological parameters except for CD8^+^ TIL density. The CD8^+^ TIL density was significantly higher in the PD-L1^+^ group than in the PD-L1- group (53/field vs. 34.5/field, respectively; *P* = 0.029). This phenomenon has also been observed in gastric adenocarcinomas [21], suggesting the possibility of an adaptive immune resistance mechanism.

Importantly, we stratified the entire cohort into four subgroups according to the expression status of PD-L1 and CD8^+^ TIL density and found that subgroup with CD8^high^ TILs and PD-L1^−^ had the best clinical outcome, whereas the subgroup with CD8^low^ TILs and PD-L1^+^ had the worst post-operative survival. These results highlight the importance of the linkage between CD8^+^ TIL density and PD-L1 expression in the tumor immune microenvironment. TILs are deemed as the fountain for cytokines such as interferon gamma (IFN-γ) [22]. IFN-γ is also likely to be a protagonist in the presence of PD-L1 on TCs [23]. Thorsson et al. analyzed immunogenomic data from 33 diverse cancer types consisted 10,000 tumors and discovered one immune subtype that was INF-γ dominant. The INF-γ cancer subtype was characterized by the highest CD8^+^ T cell level and an inferior survival time [24]. INF-γ has also been shown to induce PD-L1 expression in tumors or TILs [25], which is a potential mechanism of the high CD8^+^ TIL density in PD-L1^+^ GBC. Further validation studies are warranted to explore the relationship between CD8^+^ TILs density (and that of other immune cells, such as T_reg_) and tumor resistance via immune checkpoints [26, 27].

A model proposed by Teng et al. [28] classified tumors as type I (PD-L1^+^ TILs^+^ driving adaptive immune resistance), type II (PD-L1^−^ TIL^−^, indicating immune ignorance), type III (PD-L1^+^ TIL^−^, indicating intrinsic induction) and type IV (PD-L1^−^ TIL^+^, indicating the role of other suppressor (s) in promoting immune tolerance). However, no studies have verified the immune characteristics and clinical outcomes of GBC. Our present results support that co-evaluating CD8^+^ TILs and PD-L1 expression is significant for the prognosis of patients with GBC.

The KEYNOTE-028 trial (NCT02054806), an ongoing, multi-cohort, phase 1b trial to test the efficacy of pembrolizumab in PD-L1 positive solid tumors, released data in the 2015 European Cancer Congress^26^ on 24 patients with biliary tract cancer (BTC) who had PD-L1 positive tumors. The objective response rate (ORR) was 17% (95% CI, 5–39%), which showed promising efficacy in BTC treatment. Our results are meaningful for identifying potential immune related prognostic factors and aiding in the majorization of a prudent design for immune checkpoint therapy strategies against this tumor. First, the classification that combines PD-L1 expression and CD8^+^ TIL density potentially offer helpful clue regarding the prognosis of GBC patients. Second, CD8^+^ TIL density should be considered when administering immunotherapeutic strategies applying PD-1 and PD-L1 specific inhibitors to PD-L1 positive GBC. Third, the range of clinical outcomes observed in GBC patients with surgical resection and similar clinical stages can be partly interpreted by differences in the CD8^+^ TIL density and PD-L1 expression, as no significant differences in PD-L1 expression and CD8^+^ TIL density were observed among the different disease stages at presentation.

## Conclusion

In conclusion, our results support the clinical significance of PD-L1 expression and CD8^+^ TIL density for patients with gallbladder adenocarcinoma. Combining PD-L1 expression and CD8^+^ TIL density provides an independent prognostic factor for both PFS and OS in GBC patients.

## Additional file

Additional file 1: **Figure S1.** Representative staining patterns of FFPE GBC lesions with CD133-specific mono antibody. CD133 expression was only seen at the membranous of tumor cells. A (× 100 original magnification), B (× 200 original magnification) and C (× 400 original magnification). **Figure S2.** Kaplan-Meier analysis of progression-free survival and overall survival for different biomarkers. PD-1 expression (A and B), CD133 expression (C and D) and PD-L2 expression (E and F) were all not associated with progression-free survival and overall survival. **Figure S3.** Kaplan-Meier analysis of progression-free survival and overall survival for PD-L1+ TCs (A and B) and three patterns of PD-L1 expression (C and D). **Figure S4.** Representative staining patterns of co-expression between PD-L1 and PD-L2. A. PD-L1 expression (× 200 original magnification); B. PD-L1 expression (× 400 original magnification); C. PD-L2 expression (× 200 original magnification); D. PD-L2 expression (× 400 original magnification). **Table S1.** Clinical and pathological features of the 66 GBC patients. **Table S2.** The clinicopathological characteristic of PD-L1 expression and CD8+ TILs in gallbladder cancer. (DOCX 1126 kb)

## Abbreviations

ALP: Alkaline phosphatase
ALT: Alanine transaminase
AST: Aspartate transaminase
CA19–9: Cancer antigen 19–9
CEA: Carcino-embryonic antigen
CI: Confidence interval
CTLs: Cytotoxic T lymphocytes
DBil: Direct bilirubin
FFPE: Formalin-fixed, paraffin-embedded
GBC: Gallbladder cancer (GBS)
GGT: Glutamyl transpeptidase
HE: Hematoxylin and eosin
IHC: Immunohistochemistry
IS: Immune stroma
NA: Not applicable
OS: Overall survival
PD-L: Programmed death ligand
PFS: Progression-free survival
TAAs: Tumor associated antigens
TAMs: Tumor-associated macrophages
TBil: Total bilirubin
TCR: T cell receptor
TCs: Tumor cells
TILs: Tumor-infiltrating lymphocytes
TNM: Tumor node metastasis
