# Supplementary material for: Classification of gallbladder cancer by assessment of CD8+ TIL and PD-L1 expression
Source: BMC Cancer. 2018 Jul 28;18:766. doi: 10.1186/s12885-018-4651-8 (PMC6064069; doi:10.1186/s12885-018-4651-8)
Supplement: Supplementary file 1 — Figure S1. Representative staining patterns of FFPE GBC lesions with CD133-specific mono antibody. CD133 expression was only seen at the membranous of tumor cells. A (× 100 original magnification), B (× 200 original magnification) and C (× 400 original magnification). Figure S2. Kaplan-Meier analysis of progression-free survival and overall survival for different biomarkers. PD-1 expression (A and B), CD133 expression (C and D) and PD-L2 expression (E and F) were all not associated with progression-free survival and overall survival. Figure S3. Kaplan-Meier analysis of progression-free survival and overall survival for PD-L1+ TCs (A and B) and three patterns of PD-L1 expression (C and D). Figure S4. Representative staining patterns of co-expression between PD-L1 and PD-L2. A. PD-L1 expression (× 200 original magnification); B. PD-L1 expression (× 400 original magnification); C. PD-L2 expression (× 200 original magnification); D. PD-L2 expression (× 400 original magnification). Table S1. Clinical and pathological features of the 66 GBC patients. Table S2. The clinicopathological characteristic of PD-L1 expression and CD8+ TILs in gallbladder cancer. (DOCX 1126 kb) [file 12885_2018_4651_MOESM1_ESM.docx]

**Supplementary**


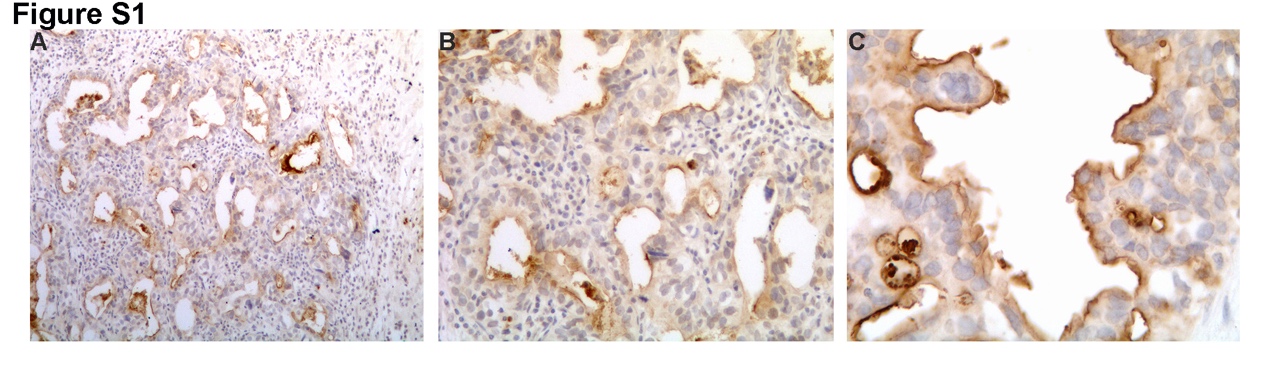


**Figure S1.** Representative staining patterns of FFPE GBC lesions with CD133-specific mono antibody. CD133 expression was only seen at the membranous of tumor cells. A (×100 original magniﬁcation), B (×200 original magniﬁcation) and C (×400 original magniﬁcation).


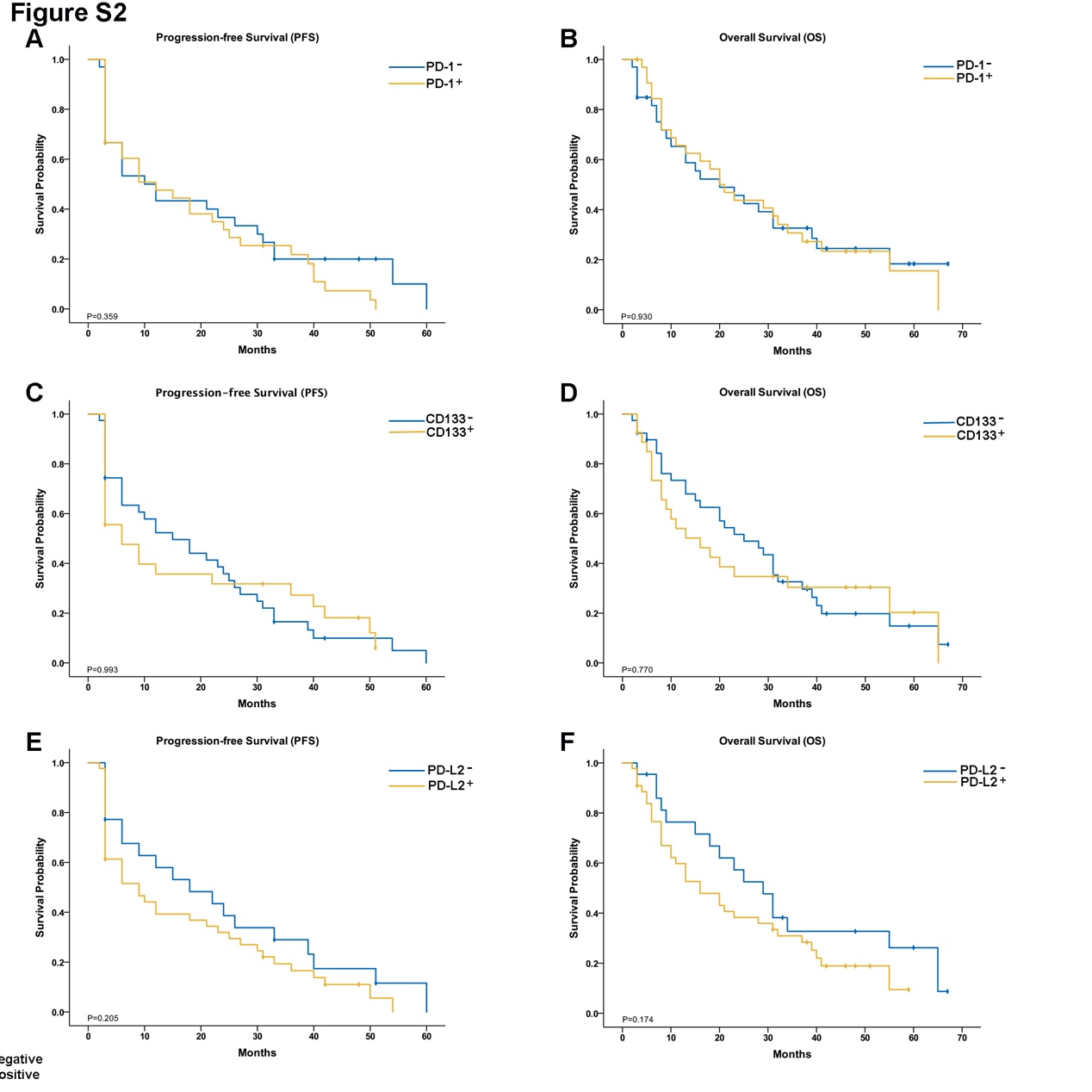


**Figure S2.** Kaplan-Meier analysis of progression-free survival and overall survival for different biomarkers. PD-1 expression (A and B), CD133 expression (C and D) and PD-L2 expression (E and F) were all not associated with progression-free survival and overall survival.


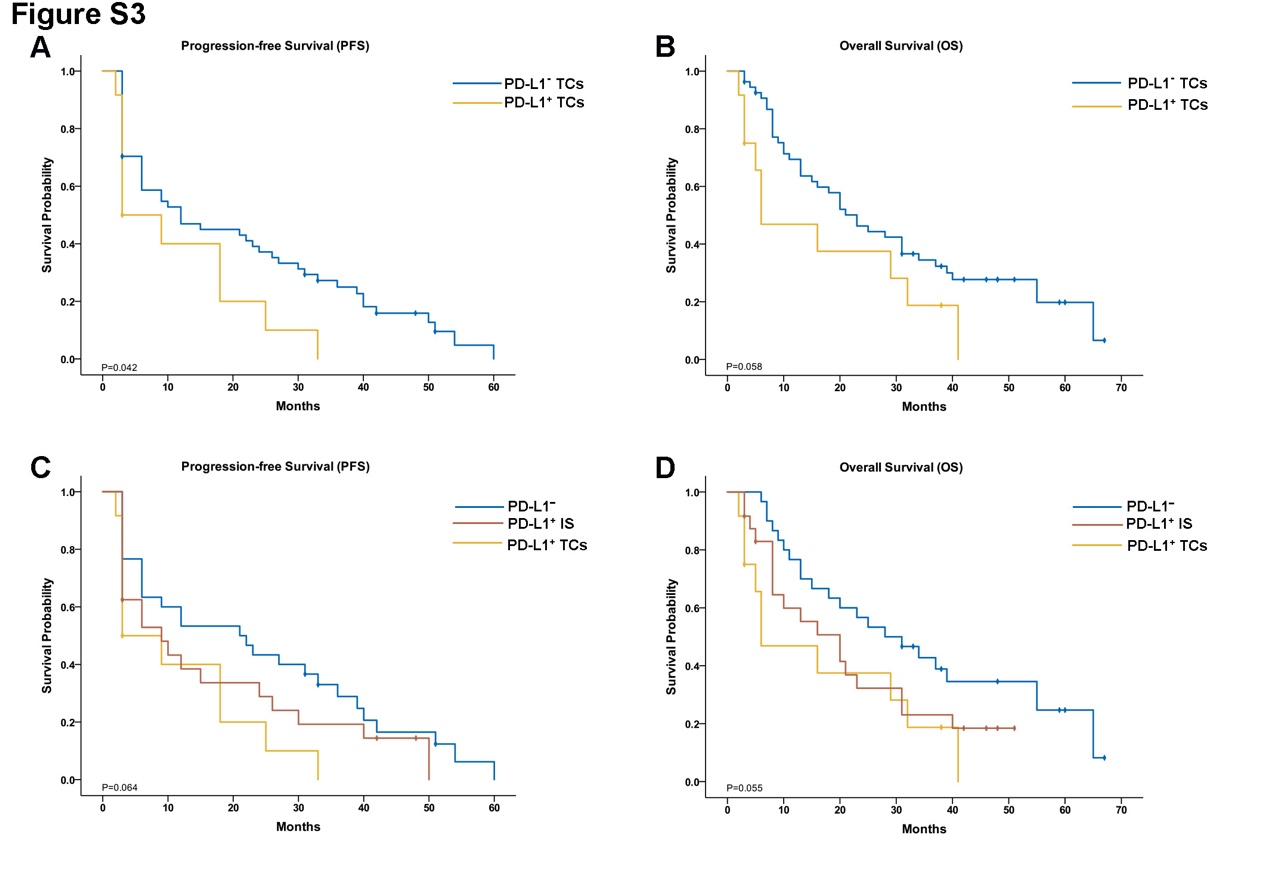


**Figure S3.** Kaplan-Meier analysis of progression-free survival and overall survival for PD-L1^+^ TCs (A and B) and three patterns of PD-L1 expression (C and D).


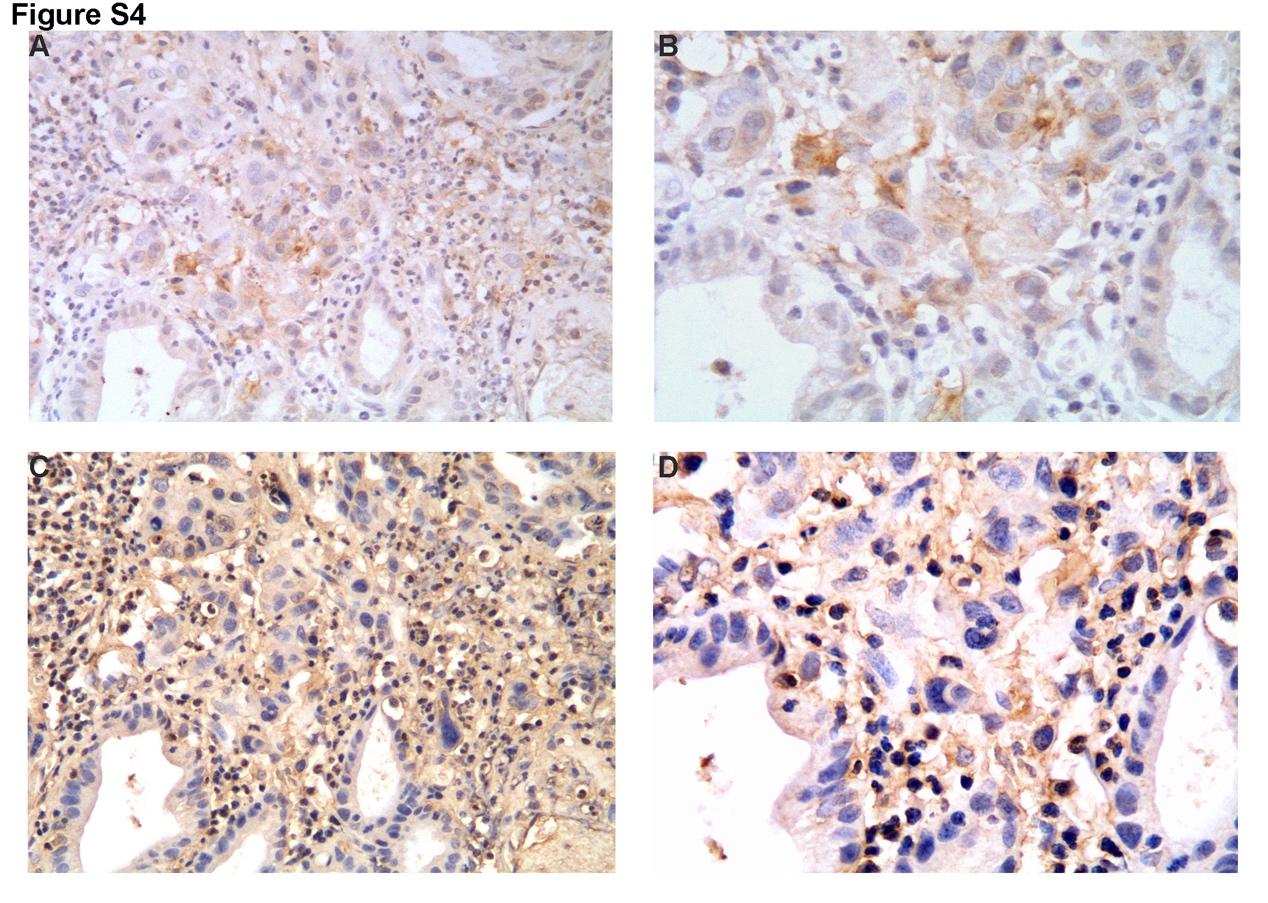


**Figure S4.** Representative staining patterns of co-expression between PD-L1 and PD-L2. A. PD-L1 expression (×200 original magniﬁcation); B. PD-L1 expression (×400 original magniﬁcation); C. PD-L2 expression (×200 original magniﬁcation); D. PD-L2 expression (×400 original magniﬁcation).

**Table S1.** Clinical and pathological features of the 66 GBC patients.

| Clinical parameters (n=66) | |
| --- | --- |
| Age, median (range) | 65 (29-81) |
| Sex (F/M) | 38 (58%)/28 (42%) |
| TNM stage  I+II  III+IV | 22 (33%)  44 (67%) |
| Risk factors  Gallstone  Diabetes  Hypertension  Cholecystitis | 36 (55%)  20 (30%)  25 (38%)  43 (65%) |
| Preoperative jaundice | 14 (21%) |
| CEA (> 5 μg/L) | 20 (30%) |
| CA19-9 (> 34 U/mL) | 35 (53%) |
| Preoperative liver dysfunction  ALT (> 40 U/L)  AST (> 35 U/L)  TBil (> 22.2 μmol/L)  DBil (> 6.8 μmol/L)  GGT (> 45 U/L)  ALP (> 135 U/L) | 15 (23%)  19 (29%)  20 (30%)  18 (27%)  20 (30%)  20 (30%) |
|  |  |
| Pathological features (n=66) | |
| Differentiation  Moderate  Poor | 28 (42%)  38 (58%) |
| Tumor size, cm (median, range) | 3.0 (0.5-7.5) |
| Immunohistochemistry  CD133^+^ in TCs, n (%)  CD8^+^ TILs density, median (range)  PD-1^+^ in TILs, n (%)  PD-L1^+^ in TCs, n (%)  PD-L1^+^ in IS, n (%)  PD-L1^+^ group, n (%)  PD-L2^+^ group, n (%) | 27 (41%)  46 (4-275)  33 (50%)  12 (18%)  24 (36%)  36 (54%)  44 (67%) |

Abbreviations: CEA: carcino-embryonic antigen, CA19-9: cancer antigen 19-9, ALT: alanine transaminase, AST: aspartate transaminase, TBil: total bilirubin, DBil: direct bilirubin, GGT: glutamyl transpeptidase, ALP: alkaline phosphatase.

**Table S2.** The clinicopathological characteristic of PD-L1 expression and CD8^+^ TILs in gallbladder cancer.

|  | PD-L1^+^ group | | | PD-L1^-^ group | *P-value | CD8^high^ TILs | CD8^low^ TILs | P-value |
| --- | --- | --- | --- | --- | --- | --- | --- | --- |
|  | PD-L1^+^ TCs (n=12) | PD-L1^+^ IS (n=24) | Overall (n=36) | (n=30) |  | (n=33) | (n=33) |  |
| Clinical parameters, n (%) |  |  |  |  |  |  |  |  |
| Age, median (range) | 64.5 (29-77) | 69.5 (41-81) | 68.5 (29-81) | 66 (48-79) | 0.694 | 64 (29-79) | 67 (45-81) | 0.108 |
| Sex (Feamle) | 6 (50%) | 15 (63%) | 21 (58%) | 17 (57%) | 0.891 | 19 (58%) | 19 (58%) | 1.000 |
| TNM stage (III + IV) | 9 (75%) | 14 (58%) | 23 (64%) | 21 (70%) | 0.600 | 19 (58%) | 25 (76%) | 0.117 |
| Risk factors  Gallstone  Diabetes  Hypertension  Cholecystitis | 6 (50%)  2 (17%)  2 (17%)  7 (58%) | 16 (67%)  7 (29%)  11 (46%)  15 (63%) | 22 (61%)  9 (25%)  13 (36%)  22 (61%) | 14 (47%)  11 (38%)  12 (40%)  21 (70%) | 0.241  0.304  0.746  0.450 | 16 (49%)  10 (30%)  12 (36%)  19 (58%) | 20 (61%)  10 (30%)  13 (39%)  24 (73%) | 0.323  1.000  0.800  0.196 |
| Preoperative jaundice | 4 (33%) | 6 (25%) | 10 (28%) | 4 (13%) | 0.153 | 6 (18%) | 8 (24%) | 0.547 |
| Radical resection | 10 (83%) | 17 (71%) | 27 (75%) | 19 (63%) | 0.304 | 24 (73%) | 22 (67%) | 0.592 |
| CEA (> 5 μg/L) | 4 (33%) | 10 (42%) | 14 (39%) | 6 (20%) | 0.096 | 7 (21%) | 13 (39%) | 0.108 |
| CA19-9 (> 34 U/mL) | 9 (75%) | 11 (46%) | 20 (56%) | 15 (50%) | 0.652 | 17 (52%) | 18 (55%) | 0.805 |
| Preoperative liver dysfunction  ALT (> 40 U/L)  AST (> 35 U/L)  TBil (> 22.2 μmol/L)  DBil (> 6.8 μmol/L)  GGT (> 45 U/L)  ALP (> 135 U/L) | 1 (8%)  3 (25%)  3 (25%)  3 (25%)  4 (33%)  4 (33%) | 8 (33%)  8 (33%)  9 (38%)  9 (38%)  8 (33%)  8 (33%) | 9 (25%)  11 (31%)  12 (33%)  12 (33%)  12 (33%)  13 (36%) | 6 (20%)  9 (30%)  8 (27%)  6 (20%)  8 (27%)  7 (23%) | 0.629  0.961  0.557  0.226  0.557  0.261 | 6 (18%)  8 (24%)  10 (30%)  8 (24%)  10 (30%)  11 (33%) | 9 (27%)  12 (36%)  10 (30%)  10 (30%)  10 (30%)  9 (27%) | 0.378  0.284  1.000  0.580  1.000  0.592 |
| Pathological features | |  |  |  |  |  |  |  |
| Differentiation (Poor) | 8 (67%) | 16 (67%) | 24 (67%) | 14 (47%) | 0.102 | 17 (52%) | 21 (64%) | 0.319 |
| Median tumor size, (range, cm) | 2.9 (0.8-7.0) | 3.0 (0.5-7.0) | 3.0 (0.5-7.0) | 3.0 (0.6-7.5) | 0.813 | 3.0 (0.5-7.5) | 2.8 (0.6-7.0) | 0.843 |
| Immunohistochemistry  CD133^+^ in TCs, n (%)  PD-1^+^ in TILs, n (%)  PD-L2^+^ in TCs, n (%)  CD8^+^ TILs density (median)  PD-L1^+^ TC, n (%)  PD-L1^+^ expression, n (%) | 5 (42%)  8 (67%)  11 (92%)  47.5 (8-148)  -  - | 11 (46%)  12 (50%)  19 (79%)  57 (9-275)  -  - | 16(44%)  20 (56%)  30 (83%)  53 (8-275)  -  - | 11 (37%)  13 (43%)  14 (47%)  34.5 (4-167)  -  - | 0.522  0.323  0.002  0.029  -  - | 14 (42%)  16 (49%)  22 (67%)  74 (47-275)  6 (18%)  21 (64%) | 13 (39%)  17 (52%)  22 (67%)  27 (4-46)  6 (18%)  15 (46%) | 0.802  0.806  1.000  <0.001  1.000  0.138 |

*P-value: comparing intergroup between PD-L1^+^ group and PD-L1^+^ group.
